# Supplementary material for: Quantitative Proteomics and Network Analysis of Differentially Expressed Proteins in Proteomes of Icefish Muscle Mitochondria Compared with Closely Related Red-Blooded Species
Source: Biology (Basel). 2022 Jul 26;11(8):1118. doi: 10.3390/biology11081118 (PMC9330239; doi:10.3390/biology11081118)
Supplement: Supplementary file 1 [file biology-11-01118-s001.zip › biology-1751198-supplementary.pdf]

biology

MDPI

Supplementary Material

Quantitative Proteomics and Network Analysis of Differentially Expressed Proteins in Proteomes of Icefish Muscle Mitochondria Compared with Closely Related Red-blooded Species

LC/LC-MS Methods

Protein was denatured by dissolving in aqueous 9 M urea buffer containing, 20 mM HEPES pH 8.0, 1 mM sodium orthovanadate, 2.5 mM sodium pyrophosphate, 1 mM β-glycerophosphate. Disulfides were reduced with 0.7 mg/mL dithiothreitol and heating at 55oC, then alkylated with 1.9 mg/mL iodoacetamide before diluting to 1 M urea with aqueous 50 mM ammonium bicarbonate. Proteolytic digestion with 0.5 ◎g Promega sequencing grade trypsin/Lys-C mix, and incubation at 37oC for 16 h.

Resulting peptides were desalted with Millipore C18 ZipTip before being re-suspended in aqueous 0.1% trifluoroacetic acid (v/v) then loaded onto an mClass nanoflow UPLC system (Waters) equipped with a nanoEaze M/Z Symmetry 100 Å C18, 5 µm trap column (180 µm x 20 mm, Waters) and a PepMap, 2 µm, 100 Å, C18 EasyNano nanocapillary column (75 ◎m x 500 mm, Thermo). The trap wash solvent was aqueous 0.05% (v:v) trifluoroacetic acid and the trapping flow rate was 15 µL/min. The trap was washed for 5 min before switching flow to the capillary column. Separation used gradient elution of two solvents: solvent A, aqueous 0.1% (v:v) formic acid; solvent B, acetonitrile containing 0.1% (v:v) formic acid. The flow rate for the capillary column was 300 nL/min and the column temperature was 40°C. The linear multi-step gradient profile was: 3-10% B over 7 mins, 10-35% B over 30 mins, 35-99% B over 5 mins and then proceeded to wash with 99% solvent B for 4 min. The column was returned to initial conditions and re-equilibrated for 15 min before subsequent injections.

The nanoLC system was interfaced with an Orbitrap Fusion Tribrid mass spectrometer (Thermo) with an EasyNano ionisation source (Thermo). Positive ESI-MS and MS2 spectra were acquired using Xcalibur software (version 4.0, Thermo). Instrument source settings were: ion spray voltage, 1,900 V; sweep gas, 0 Arb; ion transfer tube temperature; 275°C. MS1spectra were acquired in the Orbitrap with: 120,000 resolution, scan range: m/z 375-1,500; AGC target, 4e5; max fill time, 100 ms. Data dependant acquisition was performed in top speed mode using a 1 s cycle, selecting the most intense precursors with charge states >1. Easy-IC was used for internal calibration. Dynamic exclusion was performed for 50 s post precursor selection and a minimum threshold for fragmentation was set at 5e3. MS2 spectra were acquired in the linear ion trap with: scan rate, turbo; quadrupole isolation, 1.6 m/z; activation type, HCD; activation energy: 32%; AGC target, 5e3; first mass, 110 m/z; max fill time, 100 ms. Acquisitions were arranged by Xcalibur to inject ions for all available parallelizable time.

Peak lists in .raw format were loaded into PeaksStudioX for peak picking, database searching and label free quantification. Spectra were searched against the combined NCBI deposited proteins from Notothenia coriiceps(32,361 sequences; 15,554,893 residues), Chaenocephalus aceratus (223 sequences; 59,314 residues), Dissostichus mawsoni (210 sequences; 61,335 residues) and Eleginops maclovinus (193 sequences; 53,595 residues), in addition to 115 common proteomic contaminant proteins. Search criteria specified: Enzyme, trypsin; Fixed modifications, Carbamidomethyl (C); Variable modifications, Oxidation (M); Peptide tolerance, 3 ppm; MS/MS tolerance, 0.5 Da; Instrument, ESI-TRAP. Subsequent PEAKS-PTM searching was used to consider 313 UniMod listed PTMs and PEAKS-Spider searching considered single point amino acid substitutions. Data were filtered to 1% false discovery rate, as assessed against a decoy database and further

filtered to require at least two unique peptides for each protein group. Proteins that contained similar peptides and could not be differentiated based on MS/MS analysis alone were grouped to satisfy the principles of parsimony. Proteins sharing significant peptide evidence were grouped into clusters.
Proteomics protein trend across species.

Red Muscle Mitochondria

Supplementary Table S1. Proteins higher in abundance in *N. rossii*

Accession Id's	Protein Name	Relative percentage of Total Ion Area
CAC27776.1	Myosin heavy chain	33.8
XP_010777618.1	myosin heavy chain fast skeletal	66.6
AAO21697.1	alpha actin	47.9
XP_010792310.1	actin cytoplasmic 3	32.4
XP_010784415.1	alpha-actinin-3	39.5
XP_010794573.1	desmin	71.8
AAO24741.1	creatine kinase mitochondrial isoform	41.7
XP_010776201.1	titin	60.2
XP_010791686.1	alpha-actinin-2	48.2
XP_010777287.1	tropomyosin alpha-1 chain	37.3
XP_010792180.1	apolipoprotein A-I	30.9
XP_010794773.1	myomesin-2	38.0
AAC25100.1	alpha globin	60.0
AAC59671.1	alpha-1 globin	60.0
XP_010781418.1	isocitrate dehydrogenase [NADP]	32.6
XP_010784569.1	Cytochrome b-c1 complex subunit 2	64.7
XP_010767505.1	myosin regulatory light chain 2	41.6
XP_010771394.1	tropomyosin alpha-3 chain	36.9
XP_010778108.1	perilipin-3	48.8
XP_010774927.1	histone H2AX	30.8
XP_010774355.1	myomesin-1	32.4
XP_010776993.1	NADP-dependent malic enzyme	33.9
XP_010765036.1	aspartate aminotransferase	28.5
XP_010772035.1	alpha-aminoadipic semialdehyde dehydrogenase	36.3
XP_010777712.1	cytochrome b-c1 complex subunit 1	33.6
XP_010770990.1	troponin I	39.1
XP_010768648.1	nebulin	58.7
XP_010765900.1	carbonic anhydrase 1 [	49.4
XP_010788472.1	glycogen phosphorylase	30.6
XP_010771131.1	NADH dehydrogenase [ubiquinone] iron-sulphur protein 7	33.8
XP_010788098.1	heat shock protein beta 1	49.4
XP_010768865.1	cytochrome c oxidase subunit 5B	31.8
XP_010770791.1	cytochrome c oxidase subunit 4 isoform 2	60.1

XP_010766259.1	NADH dehydrogenase [ubiquinone] 1 alpha subcomplex subunit 7	39.2
XP_010792793.1	cytochrome c	42.8
XP_010767026.1	cytochrome c oxidase subunit NDUFA4	75.3
XP_010765537.1	glutaryl-CoA dehydrogenase	33.1
XP_010767938.1	fibrillin-1 isoform X1	67.5
XP_010795730.1	glutaryl-CoA dehydrogenase	34.0
XP_010776996.1	mitochondrial pyruvate carrier 2	36.7
XP_010767760.1	CDGSH iron-sulphur domain-containing protein 1	41.0
XP_010766700.1	palladin	52.7
XP_010794954.1	calcium/calmodulin-dependent protein kinase type II subunit beta	49.8
XP_010769019.1	NADH dehydrogenase [ubiquinone] 1 beta subcomplex subunit 5	33.0
XP_010773120.1	apolipoprotein O	40.0
XP_010791045.1	retinol dehydrogenase 13 isoform X1	42.1
XP_010766696.1	enoyl-CoA delta isomerase 2	42.8
XP_010783831.1	alanine aminotransferase 1	51.3
XP_010771234.1	superoxide dismutase [Mn]	42.0
XP_010795670.1	3-hydroxyisobutyrate dehydrogenase	43.5
XP_010781417.1	1-acyl-sn-glycerol-3-phosphate acyltransferase gamma	75.8
XP_010772950.1	collagen alpha-2(I) chain isoform X1	39.6
XP_010784042.1	dehydrogenase/reductase SDR family member 7C	68.9
XP_010788327.1	nebulin	57.4
XP_010778895.1	long-chain specific acyl-CoA dehydrogenase	57.8
XP_010779324.1	mitochondrial import inner membrane translocase subunit Tim21	70.4
XP_010778764.1	alanine aminotransferase 2	40.5
NP_001290228.1	fatty acid-binding protein	72.5
XP_010773093.1	Pyruvate dehydrogenase phosphatase regulatory subunit	24.9

Differentially expressed proteins in RMM in *N. rossii* are seen in Suplementary Tables 1 & 2 more abundant proteins PPI (relative ion area >25) (A) and decreased abundance of proteins (relative ion area <25) (B). Functional enrichment of the proteins showed, more abundant proteins belonging to complex I, amino acid metabolism, complex III, and muscle proteins. The less abundant proteins belonged to the functional enrichment analysis showed proteins involved in sulphur compound metabolism, organonitrogen biosynthesis and carboxylic acid metabolism.

Supplementary Table S2. Proteins lower in abundance in *N. rossii*

Accession Id's	Relative percentage of Total Ion Area	Protein Name
CAC27777.1	7.9	MyoHC-A3
XP_010765274.1	13.0	ADP/ATP translocase 2
AGN90720.1	14.8	beta-actin
XP_010776110.1	14.8	actin cytoplasmic 1
XP_010782516.1	13.2	voltage-dependent anion-selective channel protein 3
XP_010786020.1	5.5	NAD(P) transhydrogenase
AAC60356.1	2.4	fatty acid binding protein H8-isoform
XP_010772744.1	15.2	calcium-binding mitochondrial carrier protein Aralar1
XP_010773400.1	17.4	sorting and assembly machinery component 50 homolog
XP_010782695.1	9.3	ATP-dependent 6-phosphofructokinase
XP_010773584.1	20.0	mitochondrial carnitine/acylcarnitine carrier protein
XP_010771546.1	6.5	apolipoprotein B-100
XP_010768502.1	16.0	sodium/potassium-transporting ATPase subunit alpha-3
XP_010789292.1	21.0	eukaryotic initiation factor 4A-I
XP_010780086.1	6.2	NAD-dependent malic enzyme
CAL92187.1	4.0	transferrin
XP_010767902.1	9.1	hyaluronan and proteoglycan link protein 1
XP_010790586.1	3.9	KH domain-containing RNA-binding signal transduction-associated protein 1
XP_010786167.1	14.3	prothrombin
XP_010774874.1	16.3	neurofilament medium polypeptide
XP_010778213.1	15.2	pyruvate dehyrogenase phosphatase catalytic subunit 1
XP_010766309.1	16.5	cytochrome c oxidase subunit 5A isoform X2
XP_010786726.1	19.0	glutathione S-transferase kappa 1
XP_010782566.1	12.5	elongation factor 1-gamma
XP_010780100.1	13.1	stomatin protein 2
XP_010766151.1	14.9	elongation factor 1-gamma
XP_010790260.1	10.7	60S ribosomal protein L27a isoform X2
XP_010790259.1	10.7	60S ribosomal protein L27a isoform X1
XP_010772921.1	14.1	kelch protein 31
XP_010781656.1	14.1	fructose-1 6-bisphosphatase isozyme 2
XP_010793467.1	16.1	AMP deaminase 1 isoform X2
XP_010782071.1	16.5	vitellogenin-2
XP_010771562.1	13.4	glycogen debranching enzyme
XP_010773277.1	17.7	cystatin-B
XP_010782228.1	17.3	mitochondrial fission process protein 1
XP_010792412.1	9.3	stomatin protein 2
XP_010791811.1	8.4	60S ribosomal protein L23a
XP_010795420.1	18.5	synaptic vesicle membrane protein VAT-1 homolog
XP_010774286.1	18.6	kelch protein 41b
XP_010787572.1	18.3	methenyltetrahydrofolate synthase domain-containing protein isoform X1
XP_010778366.1	17.7	ATP-dependent Clp protease proteolytic subunit
XP_010784742.1	6.7	calpain small subunit 1
XP_010779525.1	7.5	aconitate hydratase

XP_010773293.1	9.0	histone H1
XP_010776100.1	17.3	mitochondrial pyruvate carrier 1
XP_010792543.1	6.7	flotillin-1
XP_010789836.1	14.4	60S ribosomal protein L13
XP_010790338.1	10.2	epoxide hydrolase 1
XP_010793737.1	16.9	fatty-acid amide hydrolase 1
XP_010792494.1	10.9	malonyl-CoA decarboxylase
XP_010792115.1	12.0	glutathione S-transferase A
XP_010766317.1	13.2	malate dehydrogenase
XP_010796013.1	1.4	myosin heavy chain fast
XP_010782096.1	6.8	14-3-3 protein beta/alpha-1
AAK07470.1	2.6	long chain fatty acyl CoA synthetase
XP_010780333.1	4.9	26S protease regulatory subunit 4 isoform X1
XP_010766277.1	20	Stress-70 protein mitochondrial-like
XP_010772138.1	24.6	ATP synthase subunit O
XP_010779232.1	23.1	ATP synthase subunit g mitochondrial
XP_010779868.1	22.2	ATP synthase subunit alpha mitochondrial
XP_010765728.1	24.7	ATP synthase subunit beta mitochondrial
XP_010778067.1	18.3	ATP synthase subunit gamma mitochondrial isoform X1
XP_010786327.1	23	ATP synthase F(0) complex subunit B1 mitochondrial
XP_010775450.1	18.8	ATP synthase subunit delta mitochondrial
XP_010784438.1	23.5	ADP/ATP translocase 3
XP_010783746.1	15.9	60S ribosomal protein L23
XP_010793356.1	17.2	Ubiquinone biosynthesis protein COQ9

Supplementary Table S3. Proteins higher in abundance in *C. rastrospinosus*

Accession Id's	Relative percentage of Total Ion Area	Protein Name
XP_010765274.1	36.5	ADP/ATP translocase 2
XP_010774339.1	39.3	ADP/ATP translocase 1
XP_010782516.1	43.2	voltage-dependent anion-selective channel protein 3
XP_010777287.1	30.4	tropomyosin alpha-1 chain isoform X1
AAC60356.1	37.2	fatty acid binding protein H8-isoform
XP_010770006.1	37.2	NADH dehydrogenase [ubiquinone] iron-sulfur protein 2
XP_010772744.1	42	calcium-binding mitochondrial carrier protein Aralar1
XP_010773400.1	37.3	sorting and assembly machinery component 50 homolog
XP_010790716.1	33.5	NADH dehydrogenase [ubiquinone] flavoprotein 1
XP_010789886.1	33.7	cytochrome c1 heme protein mitochondrial
XP_010774927.1	42.5	histone H2AX
XP_010794136.1	34.4	ATP synthase subunit g
XP_010785217.1	36.2	NADH dehydrogenase [ubiquinone] iron-sulfur protein 3
XP_010768357.1	32.8	calcium-binding mitochondrial carrier protein Aralar1
XP_010773584.1	36.2	mitochondrial carnitine/acylcarnitine carrier protein
XP_010768822.1	38	NADH dehydrogenase [ubiquinone] flavoprotein 2
XP_010792996.1	40.5	histone H4
XP_010766804.1	32	sideroflexin-1
XP_010794867.1	36.9	sodium/potassium-transporting ATPase subunit alpha-1
XP_010769976.1	44.2	myosin-binding protein H
XP_010765220.1	38.3	NADH dehydrogenase [ubiquinone] 1 alpha subcomplex subunit 12
XP_010789215.1	45.7	NADH dehydrogenase [ubiquinone] 1 alpha subcomplex subunit 9
XP_010785201.1	31.9	titin
XP_010789292.1	43.4	eukaryotic initiation factor 4A-I
XP_010766730.1	37.5	ATP synthase subunit d
XP_010773903.1	50.1	cytochrome b-c1 complex subunit Rieske
XP_010766290.1	42.6	elongation factor Tu
XP_010778686.1	38.8	isocitrate dehydrogenase [NAD] subunit alpha
CAL92187.1	40.5	transferrin
XP_010780431.1	38.9	cytochrome b-c1 complex subunit 8
XP_010772963.1	38.2	NADH dehydrogenase [ubiquinone] 1 beta subcomplex subunit 4
XP_010768865.1	35.4	cytochrome c oxidase subunit 5B
XP_010775725.1	43	NADH dehydrogenase [ubiquinone] 1 alpha subcomplex subunit 5
XP_010783386.1	38.7	EH domain-containing protein 2 isoform X2
XP_010784582.1	35.8	NADH dehydrogenase [ubiquinone] 1 alpha subcomplex subunit 9
XP_010793340.1	46.4	obg ATPase 1
XP_010778644.1	36.9	NADH dehydrogenase [ubiquinone] 1 beta subcomplex subunit 3
XP_010766309.1	46.4	cytochrome c oxidase subunit 5Al isoform X2
XP_010766259.1	30.1	NADH dehydrogenase [ubiquinone] 1 alpha subcomplex subunit 7
XP_010770879.1	40.8	NADH dehydrogenase [ubiquinone] 1 alpha subcomplex subunit 8
XP_010771148.1	30.2	troponin C
XP_010786726.1	45	glutathione S-transferase kappa 1
XP_010782566.1	45.1	elongation factor 1-gamma

XP_010780100.1	43.6	stomatin protein 2
XP_010766151.1	40	elongation factor 1-gamma
XP_010790260.1	38.9	60S ribosomal protein L27a isoform X2
XP_010790259.1	38.9	60S ribosomal protein L27a isoform X1
XP_010792329.1	39.6	NADH dehydrogenase [ubiquinone] 1 beta subcomplex subunit 6
XP_010782228.1	49.4	mitochondrial fission process protein 1
XP_010792412.1	47.1	stomatin protein 2
XP_010791811.1	37.2	60S ribosomal protein L23a
XP_010784618.1	37.8	NADH dehydrogenase [ubiquinone] 1 alpha subcomplex subunit 6
XP_010779525.1	72.8	aconitate hydratase
XP_010773293.1	57.3	histone H1
XP_010776100.1	45.7	mitochondrial pyruvate carrier 1
XP_010789836.1	43.3	60S ribosomal protein L13
XP_010766317.1	42.1	malate dehydrogenase
XP_010786327.1	36	ATP synthase F(0) complex subunit B1 mitochondrial
XP_010765152.1	36.9	NADH dehydrogenase [ubiquinone] 1 alpha subcomplex subunit 10
XP_010788090.1	42	complement component 1 Q subcomponent-binding
XP_010774413.1	44.8	adenylosuccinate synthetase isozyme 1
XP_010787501.1	37.1	60S ribosomal protein L5
XP_010791295.1	39.4	pyruvate dehydrogenase (acetyl-transferring) kinase isozyme 2
XP_010786834.1	37.2	60S ribosomal protein L18
XP_010784234.1	39.5	NADH-cytochrome b5 reductase 3
XP_010764848.1	37.1	moesin isoform X1
XP_010792965.1	39.4	40S ribosomal protein S4
XP_010792689.1	42.5	cytochrome b-c1 complex subunit 1
XP_010782003.1	34.2	carnitine O-palmitoyltransferase 1
XP_010764516.1	69.8	nucleolar protein 56
XP_010783746.1	37	60S ribosomal protein L23
XP_010788817.1	33.3	phosphoglucomutase-1
XP_010764118.1	33.6	probable 2-oxoglutarate dehydrogenase E1 component DHKTD1
XP_010766620.1	35	proteasome subunit alpha type-5
XP_010788586.1	33.5	nucleolin
XP_010792044.1	41.2	isocitrate dehydrogenase [NAD] subunit beta isoform X2
XP_010785528.1	31.6	annexin A6 isoform X1
XP_010785529.1	31.6	annexin A6 isoform X2
XP_010793356.1	42.1	ubiquinone biosynthesis protein COQ9
XP_010788727.1	35.4	heat shock protein HSP 90-alpha 1
XP_010786454.1	36.9	heat shock protein 75 kDa
XP_010776005.1	39.7	transmembrane emp24 domain-containing protein 10
XP_010795693.1	33.3	60S ribosomal protein L4
XP_010767141.1	30.9	voltage-dependent anion-selective channel protein 2
XP_010787333.1	39.1	voltage-dependent anion-selective channel protein 1
XP_010783785.1	38.5	40S ribosomal protein S12
XP_010779868.1	31.7	ATP synthase subunit alpha
XP_010779103.1	33.4	60S ribosomal protein L12 isoform X1
XP_010779104.1	33.4	60S ribosomal protein L12 isoform X2
XP_010788657.1	27.9	60S ribosomal protein L7a
XP_010778828.1	34.2	26S protease regulatory subunit 8

XP_010777506.1	30.4	NADH-ubiquinone oxidoreductase 75 kDa
XP_010774792.1	34.1	60S ribosomal protein L18a
XP_010772138.1	29.1	ATP synthase subunit O
XP_010782878.1	34.4	40S ribosomal protein S18
XP_010774669.1	36.1	voltage-dependent anion-selective channel protein 2
XP_010772721.1	35.6	succinyl-CoA ligase [ADP-forming] subunit beta
XP_010788571.1	30.6	T-complex protein 1 subunit gamma
XP_010773777.1	38.3	40S ribosomal protein S16 isoform X1
XP_010769328.1	31.9	phosphoglycerate mutase 2
XP_010795246.1	35.8	elongation factor 1-beta
XP_010773724.1	39.1	prohibitin
XP_010777155.1	32.8	thioredoxin-related transmembrane protein 1
XP_010790696.1	36.6	mitochondrial import receptor subunit TOM40 homolog
XP_010774042.1	27.6	FK506-binding protein 1
XP_010795081.1	30	annexin A4
XP_010765060.1	33.9	60S ribosomal protein L21
XP_010774335.1	35.6	fatty acid-binding protein
XP_010774833.1	36.2	eukaryotic translation initiation factor 2 subunit 1
XP_010789111.1	29.3	glyceraldehyde-3-phosphate dehydrogenase
XP_010766874.1	34.7	40S ribosomal protein S20
XP_010779594.1	53.4	proteasome subunit alpha type-2
XP_010773596.1	31.8	succinate dehydrogenase [ubiquinone] iron-sulfur subunit
XP_010776147.1	30.6	40S ribosomal protein S3
XP_010795570.1	91.7	ryanodine receptor 1
XP_010773129.1	49.5	transferrin receptor protein 1
XP_010786813.1	42.5	40S ribosomal protein S9
XP_010777993.1	45.8	ATP synthase subunit s
XP_010765492.1	44.8	UTP--glucose-1-phosphate uridylyltransferase
XP_010786020.1	29.6	NAD(P) transhydrogenase mitochondrial-like
XP_010782086.1	24	Sarcolemmal membrane-associated protein
XP_010773292.1	27.3	Dihydrolipoyllysine-residue acetyltransferase component of pyruvate dehydrogenase
XP_010766277.1	33.7	Stress-70 protein
XP_010775450.1	28.4	ATP synthase subunit delta mitochondrial
XP_010781933.1	27.9	Apolipoprotein B-100-like partial
XP_010783388.1	40.7	26S proteasome non-ATPase regulatory subunit 8

PPI network for differentially expressed proteins in RMM in *C. rastrospinosus* are seen in **Supplementary Table 3 & 4**.The more abundant proteins (relative ion area >25) (A) and decreased abundance of proteins (relative ion area <25 (B). Functional enrichment of the proteins showed, more abundant proteins belonging to SRP-dependent co-translational, complex I, complex III, and complex V and mitochondrial transport. The less abundant protein functional enrichment analysis showed proteins involved in glycogen metabolism, regulation of NADP metabolic process, sarcomere organization, and isocitrate metabolism.

Supplementary Table S4. Proteins lower in abundance in *C. rastrospinosus*

Accession Id's	Relative percent-age of Total Ion Area	Protein Name
CAC27776.1	1.4	myosin heavy chain
CAC27777.1	1.9	MyoHC-A3
AAO21697.1	8.7	alpha actin
XP_010784415.1	13.3	alpha-actinin-3
XP_010794573.1	8.6	desmin
AAO24741.1	2.3	creatine kinase mitochondrial isoform
XP_010772488.1	1.4	creatine kinase S-type
XP_010781940.1	14.0	aconitate hydratase
XP_010793425.1	0.7	myosin-7 isoform X1
XP_010791227.1	12.1	myosin-binding protein C fast-type isoform X2
XP_010776201.1	11.1	titin
XP_010792180.1	15.3	apolipoprotein A-I
XP_010794773.1	10.5	myomesin-2
XP_010785535.1	13.1	keratin type II cytoskeletal 8
AAC25100.1	0.8	alpha globin
AAC59671.1	0.8	alpha-1 globin
XP_010781418.1	17.2	isocitrate dehydrogenase [NADP]
XP_010784569.1	12.4	cytochrome b-c1 complex subunit 2 isoform X1
XP_010771394.1	11.0	tropomyosin alpha-3 chain
XP_010778108.1	11.1	perilipin-3
XP_010774355.1	8.8	myomesin-1
XP_010776993.1	16.0	NADP-dependent malic enzyme
XP_010765036.1	21.7	aspartate aminotransferase cytoplasmic
XP_010772035.1	20.0	alpha-aminoadipic semialdehyde dehydrogenase
XP_010777712.1	6.8	cytochrome b-c1 complex subunit 1
XP_010767025.1	11.5	plectin isoform X1
XP_010791816.1	15.3	fructose-bisphosphate aldolase C-B
XP_010768648.1	5.2	nebulin
XP_010765900.1	10.9	carbonic anhydrase 1
XP_010788472.1	10.9	glycogen phosphorylase
XP_010780086.1	17.0	NAD-dependent malic enzyme
XP_010773075.1	6.6	M-protein striated muscle
XP_010794764.1	10.1	fibronectin
XP_010792793.1	16.1	cytochrome c
XP_010767026.1	12.6	cytochrome c oxidase subunit NDUFA4
XP_010782071.1	12.9	vitellogenin-2
XP_010771562.1	9.2	glycogen debranching enzyme
XP_010787466.1	14.0	methylcrotonoyl-CoA carboxylase subunit alpha isoform X1
XP_010788355.1	19.3	glycogen phosphorylase
XP_010771234.1	11.5	superoxide dismutase [Mn]
XP_010791177.1	17.0	glycerol-3-phosphate dehydrogenase
XP_010770992.1	12.5	troponin I
XP_010773093.1	15.4	pyruvate dehydrogenase phosphatase regulatory subunit

XP_010785995.1	2.1	band 3 anion transport protein
XP_010786435.1	21.3	heme oxygenase 2
XP_010784864.1	20.5	Troponin T fast skeletal muscle isoforms-like isoform X1 to X3
XP_010765699.1	20.3	PDZ and LIM domain protein 7
XP_010772035.1	20	Alpha-aminoadipic semialdehyde dehydrogenase
XP_010791917.1	23.3	Creatine kinase M-type
XP_010769508.1	21.1	Ubiquitin carboxyl-terminal hydrolase 5 isoform X1 & X2
XP_010784587.1	21.5	Methylmalonyl-CoA mutase

Supplementary Table S5. Proteins more in abundance in *C. gunnari*

Accession Id's	Relative percentage of Total Ion Area	Protein Name
NP_001290213.1	69.7	myosin heavy chain
XP_010765274.1	44.3	ADP/ATP translocase 2
XP_010781933.1	65.6	apolipoprotein B-100
NP_001290222.1	33.9	long-chain-fatty-acid--CoA ligase 1
XP_010782516.1	31.6	voltage-dependent anion-selective channel protein 3
XP_010786020.1	59.2	NAD(P) transhydrogenase
XP_010788987.1	30.8	titin
AAC60356.1	34.8	fatty acid binding protein H8-isoform
XP_010773400.1	36.4	sorting and assembly machinery component 50 homolog
XP_010794136.1	31.9	ATP synthase subunit g
XP_010777712.1	32.5	cytochrome b-c1 complex subunit 1
XP_010768357.1	36.4	calcium-binding mitochondrial carrier protein Aralar1
XP_010773584.1	33.9	mitochondrial carnitine/acylcarnitine carrier protein
XP_010771546.1	83.3	apolipoprotein B-100
XP_010766804.1	34.0	sideroflexin-1
XP_010767902.1	55.7	hyaluronan and proteoglycan link protein 1
XP_010772963.1	27.7	NADH dehydrogenase [ubiquinone] 1 beta subcomplex subunit 4
XP_010786167.1	29.3	prothrombin
XP_010770879.1	29.8	NADH dehydrogenase [ubiquinone] 1 alpha subcomplex subunit 8
XP_010766151.1	31.1	elongation factor 1-gamma
XP_010790260.1	35.6	60S ribosomal protein L27a isoform X2
XP_010790259.1	35.6	60S ribosomal protein L27a isoform X1
XP_010771562.1	31.8	glycogen debranching enzyme
XP_010774059.1	33.7	AFG3 protein 2
XP_010776936.1	43.8	60S ribosomal protein L28
XP_010790338.1	37.1	epoxide hydrolase 1
XP_010766317.1	27.8	malate dehydrogenase
XP_010786327.1	29.5	ATP synthase F(0) complex subunit B1
XP_010778067.1	30.1	ATP synthase subunit gamma X1
XP_010773598.1	35.0	ATPase family AAA domain-containing protein 3A
XP_010770997.1	42.4	dynamin-1 protein

XP_010768163.1	64.7	adenylate kinase 4
XP_010793736.1	39.3	inter-alpha-trypsin inhibitor heavy chain H4
XP_010794434.1	34.2	ATP-dependent 6-phosphofructokinase
XP_010767276.1	31.0	ATP-binding cassette sub-family B member 7
XP_010765728.1	30.8	ATP synthase subunit beta
XP_010774792.1	28.9	60S ribosomal protein L18a
XP_010772138.1	32.9	ATP synthase subunit O
XP_010779169.1	35.6	NADH dehydrogenase [ubiquinone] 1 beta subcomplex subunit 8
XP_010780333.1	81.4	26S protease regulatory subunit 4 isoform X1
XP_010780334.1	81.4	26S protease regulatory subunit 4 isoform X2
XP_010765060.1	39.8	60S ribosomal protein L21
XP_010772504.1	39.8	voltage-dependent anion-selective channel protein 2 isoform X2
XP_010775450.1	29.4	ATP synthase subunit delta
XP_010771726.1	30.4	cytochrome c oxidase subunit 4 isoform 1 isoform X1
XP_010771732.1	30.4	cytochrome c oxidase subunit 4 isoform 1 isoform X2
XP_010790855.1	34.2	obscurin isoform X3
XP_010790853.1	34.2	obscurin isoform X1
XP_010790854.1	34.2	obscurin isoform X2
XP_010781840.1	40.8	transitional endoplasmic reticulum ATPase
XP_010780029.1	52.3	ATP-binding cassette sub-family F member 2
XP_010773228.1	48.1	26S proteasome non-ATPase regulatory subunit 6
XP_010770797.1	63.5	fragile X mental retardation syndrome-related protein 2
XP_010768595.1	37.7	stress-70 protein
XP_010790499.1	29.6	60S ribosomal protein L35
XP_010786327.1	29.5	ATP synthase F(0) complex subunit B1 mitochondrial
XP_010775450.1	29.4	ATP synthase subunit delta
XP_010784438.1	29.0	ADP/ATP translocase 3
XP_010786167.1	29.3	Prothrombin partial
XP_010774792.1	28.9	60S ribosomal protein L18a-like
XP_010766730.1	28.7	ATP synthase subunit d
XP_010766317.1	27.8	Malate dehydrogenase
XP_010766309.1	27.8	Cytochrome c oxidase subunit 5A
XP_010783746.1	26.7	60S ribosomal protein L23
XP_010773724.1	24.5	Prohibitin
XP_010793356.1	23	Ubiquinone biosynthesis protein

The differentially expressed proteins in RMM in *C. gunnari* are seen in **Supplementary Table 5 & 6.** The more abundant proteins (relative ion area >25) (A) and decreased abundance of proteins (relative ion area <25) (B). Functional enrichment of the proteins showed, more abundant proteins belonging NADH metabolic process, ATP synthase coupled transport, cytoskeleton organization, and transmembrane transport and the less abundant proteins, the functional enrichment analysis showed proteins involved in scavenging free radicals, complex III, sarcomere organization, and muscle filament sliding

Supplementary Table S6. Proteins less in abundance in *C. gunnari*

Accession Id's	Relative percentage of Total Ion Area	Protein Name
AGN90720.1	12.1	beta-actin
XP_010776110.1	12.1	actin cytoplasmic 1

XP_010794573.1	9.9	desmin
XP_010772488.1	4.8	creatine kinase S-type
XP_010781940.1	14.0	aconitate hydratase
XP_010793425.1	3.9	myosin-7 isoform X1
AAC25100.1	0.5	alpha globin
AAC59671.1	0.5	alpha-1 globin
XP_010767505.1	8.1	myosin regulatory light chain 2
XP_010771394.1	16.1	tropomyosin alpha-3 chain
XP_010778108.1	4.5	perilipin-3
XP_010776993.1	9.8	NADP-dependent malic enzyme
XP_010765036.1	15.4	aspartate aminotransferase
XP_010772035.1	14.7	alpha-aminoadipic semialdehyde dehydrogenase
XP_010768975.1	14.8	collagen alpha-1(I) chain
XP_010785475.1	10.5	titin
XP_010791816.1	11.8	fructose-bisphosphate aldolase C-B
XP_010768648.1	15.2	nebulin
CAL92189.1	5.5	transferrin
NP_001290225.1	5.5	serotransferrin precursor
XP_010765900.1	19.6	carbonic anhydrase 1
XP_010788472.1	14.4	glycogen phosphorylase
XP_010772394.1	14.7	titin
XP_010766290.1	12.4	elongation factor Tu
XP_010778686.1	12.6	isocitrate dehydrogenase [NAD] subunit alpha
XP_010768865.1	12.2	cytochrome c oxidase subunit 5B
XP_010775725.1	22.1	NADH dehydrogenase [ubiquinone] 1 alpha subcomplex subunit 5
XP_010782377.1	3.7	calsequestrin-1
XP_010770791.1	7.7	cytochrome c oxidase subunit 4 isoform 2
XP_010792793.1	12.5	cytochrome c
XP_010771148.1	9.9	troponin C
XP_010786726.1	15.5	glutathione S-transferase kappa 1
XP_010767938.1	9.0	fibrillin-1 isoform X1
XP_010784864.1	12.7	troponin T fast skeletal muscle isoforms isoform X4
XP_010767760.1	11.5	CDGSH iron-sulphur domain-containing protein 1
XP_010765498.1	10.4	ornithine aminotransferase
XP_010790914.1	13.5	flotillin-2 isoform X1
XP_010776148.1	13.2	enoyl-CoA delta isomerase 1
XP_010795570.1	3.2	ryanodine receptor 1
XP_010779324.1	10.0	mitochondrial import inner membrane translocase subunit Tim21
XP_010774135.1	6.2	clathrin heavy chain 1
XP_010765900.1	19.6	Carbonic anhydrase 1
XP_010784415.1	18.7	Alpha-actinin-3
XP_010772948.1	18.6	probable acyl-CoA dehydrogenase 6
XP_010783999.1	15.4	Peroxiredoxin-5
XP_010771394.1	16.1	Troponin alpha-3 chain-like
XP_010779541.1	15.8	Short-chain specific acyl-CoA dehydrogenase
XP_010791917.1	16.3	Creatine kinase M-type
XP_010791177.1	16.9	Glycerol-3-phosphate dehydrogenase
XP_010791064.1	17.1	electron transfer flavoprotein subunit beta

XP_010775191.1	17.1	1 4-alpha-glucan-branching enzyme
XP_010769508.1	18	Ubiquitin carboxyl-terminal hydrolase 5 isoform X1 & X2
XP_010784587.1	18	Methylmalonyl-CoA mutase

White Muscle Mitochondria (WMM)

Supplementary Table S7. Proteins more in abundance in *N. rossii*

Accession Id's	Relative percentage of Total Ion Area	Protein Name
CAC27776.1	49.0	myosin heavy chain
XP_010768010.1	44.1	titin
XP_010785479.1	40.6	titin
XP_010791917.1	37.8	creatine kinase M-type
XP_010778896.1	41.0	myosin light chain 1/3
XP_010782377.1	41.0	calsequestrin-1
XP_010792180.1	54.5	apolipoprotein A-I
XP_010772488.1	78.2	creatine kinase S-type
XP_010787448.1	54.7	keratin type I cytoskeletal 19
AAC25100.1	79.7	alpha globin
AAC59671.1	79.7	alpha-1 globin
XP_010794476.1	53.1	betaine--homocysteine S-methyltransferase 1
XP_010788362.1	38.3	annexin A1
XP_010781921.1	49.0	histidine-rich glycoprotein
XP_010764663.1	35.3	myozenin-1
XP_010764532.1	45.1	ryanodine receptor 1
XP_010771614.1	29.0	tropomyosin alpha-1 chain isoform X3
XP_010773841.1	35.1	40S ribosomal protein S3a
XP_010794954.1	9.5	calcium/calmodulin-dependent protein kinase type II subunit beta
AAC60372.1	87.5	beta-globin
XP_010781066.1	41.2	aspartate aminotransferase
XP_010783999.1	47.5	peroxiredoxin-5
XP_010765488.1	54.8	malate dehydrogenase
XP_010778325.1	40.2	haemoglobin subunit beta-C
XP_010788340.1	40.7	hemopexin
XP_010775215.1	62.6	collagen alpha-3(VI) chain
XP_010791819.1	60.6	T-complex protein 1 subunit theta isoform X1
XP_010785995.1	64.3	band 3 anion transport protein
XP_010771898.1	51.4	fibrinogen alpha chain
XP_010778322.1	80.6	haemoglobin subunit zeta
XP_010791578.1	43.2	40S ribosomal protein S11
XP_010778161.1	81.2	transmembrane protease serine 2
XP_010776993.1	95.0	NADP-dependent malic enzyme
CAL92189.1	36.3	transferrin
NP_001290225.1	36.3	serotransferrin precursor
XP_010778035.1	58.2	succinate dehydrogenase [ubiquinone] flavoprotein

The differentially expressed proteins in WMM in *N. rossii* are seen in **supplementary Tables 7 & 8.** The more abundant proteins (relative ion area >25) (A) and decreased abundance of proteins (relative ion area <25) (B). Functional enrichment of the proteins showed, more abundant proteins belonging to hydrogen peroxide metabolism, sarcomere organisation, platelet degranulation and the less abundant proteins had the functional enrichment analysis showed proteins involved in ATP synthase coupled proton transport, cristae formation, pyruvate metabolism, transmembrane transport.

Table S8. Proteins less in abundance in *N. rossii*

Accession Id's	Relative percentage of Total Ion Area	Protein Name
XP_010766216.1	11.0	pyruvate kinase PKM
XP_010781933.1	4.2	apolipoprotein B-100
XP_010779868.1	19.0	ATP synthase subunit alpha
XP_010794573.1	15.8	desmin
XP_010770965.1	1.0	myosin regulatory light chain 2
XP_010794434.1	7.1	ATP-dependent 6-phosphofructokinase
XP_010785886.1	22.3	nucleoside diphosphate kinase B
XP_010769976.1	8.4	myosin-binding protein H
XP_010764264.1	16.0	elongation factor 2
XP_010774339.1	20.6	ADP/ATP translocase 1
XP_010788727.1	10.9	heat shock protein HSP 90-alpha 1
XP_010782071.1	9.6	vitellogenin-2
XP_010774413.1	9.7	adenylosuccinate synthetase isozyme 1
XP_010766317.1	2.8	malate dehydrogenase
XP_010777526.1	17.7	nebulin
XP_010789292.1	5.9	eukaryotic initiation factor 4A-I
XP_010793340.1	11.8	obg ATPase 1
XP_010792965.1	7.5	40S ribosomal protein S4
XP_010766151.1	5.7	elongation factor 1-gamma
XP_010773292.1	7.9	dihydrolipoyllysine-residue acetyltransferase component of pyruvate dehydrogenase complex
XP_010771231.1	9.8	T-complex protein 1 subunit alpha
XP_010768032.1	10.0	40S ribosomal protein SA isoform X2
XP_010781656.1	9.8	fructose-1 6-bisphosphatase isozyme 2
XP_010774286.1	4.2	kelch protein 41b
XP_010783756.1	6.8	40S ribosomal protein S2
XP_010785174.1	8.5	T-complex protein 1 subunit delta
XP_010779161.1	15.6	60S ribosomal protein L11 isoform X2
XP_010776310.1	4.2	60S ribosomal protein L9
XP_010794136.1	18.7	ATP synthase subunit g
XP_010793781.1	12.7	phosphoglycerate kinase
XP_010790499.1	3.4	60S ribosomal protein L35
XP_010782543.1	7.0	60S ribosomal protein L6
XP_010765728.1	17.5	ATP synthase subunit beta
XP_010779184.1	12.4	elongation factor 1-alpha
XP_010771942.1	12.3	vitellogenin-1
XP_010785200.1	2.7	palladin
XP_010791484.1	14.8	60S ribosomal protein L10a

AAL99930.1	0.0	IgM heavy chain secretory form
BAV53298.1	2.6	cytochrome c oxidase subunit II
XP_010782566.1	7.1	elongation factor 1-gamma
XP_010778067.1	13.4	ATP synthase subunit gamma
XP_010793954.1	15.8	plasma membrane calcium-transporting ATPase 2
XP_010789836.1	8.2	60S ribosomal protein L13
XP_010778226.1	5.5	succinyl-CoA ligase [ADP/GDP-forming] subunit alpha
XP_010794954.1	9.5	calcium/calmodulin-dependent protein kinase type II subunit beta
XP_010771562.1	16.3	glycogen debranching enzyme
XP_010786327.1	19.2	ATP synthase F(0) complex subunit B1
XP_010787537.1	9.1	40S ribosomal protein S8
XP_010784438.1	7.8	ADP/ATP translocase 3
XP_010772744.1	8.9	calcium-binding mitochondrial carrier protein Aralar1
XP_010788735.1	15.4	glucose-6-phosphate isomerase
XP_010778188.1	10.0	sarcalumenin
AAC60356.1	7.7	fatty acid binding protein H8-isoform
XP_010795693.1	3.8	60S ribosomal protein L4
XP_010789111.1	18.0	glyceraldehyde-3-phosphate dehydrogenase
XP_010764689.1	3.5	ATP-dependent RNA helicase DDX3X
XP_010791686.1	12.1	alpha-actinin-2
XP_010765741.1	19.1	heat shock 70 kDa protein
XP_010786813.1	12.5	40S ribosomal protein S9
XP_010791811.1	5.6	60S ribosomal protein L23a
XP_010776087.1	15.9	NAD(P) transhydrogenase
XP_010765274.1	12.1	ADP/ATP translocase 2
XP_010767141.1	15.6	voltage-dependent anion-selective channel protein 2
XP_010781251.1	8.2	26S protease regulatory subunit 6A
XP_010770361.1	6.9	60S ribosomal protein L7
XP_010764414.1	1.5	troponin C
XP_010796172.1	14.1	40S ribosomal protein S19
XP_010766874.1	9.1	40S ribosomal protein S20
XP_010768357.1	9.8	calcium-binding mitochondrial carrier protein Aralar1
XP_010766286.1	3.5	glycogen [starch] synthase
XP_010795485.1	2.6	fragile X mental retardation syndrome-related protein 1 isoform X3
XP_010784570.1	11.7	cytochrome b-c1 complex subunit 2 isoform X2
XP_010765339.1	13.0	alpha-enolase
XP_010769508.1	9.3	ubiquitin carboxyl-terminal hydrolase 5 isoform X2
XP_010772138.1	16.1	ATP synthase subunit O
XP_010783741.1	17.0	40S ribosomal protein S5
XP_010773196.1	5.9	betaine--homocysteine S-methyltransferase 1
XP_010776714.1	13.0	40S ribosomal protein S25
XP_010795528.1	17.7	decorin
XP_010795081.1	3.3	annexin A4
XP_010769990.1	9.2	heat shock 70 kDa protein 6
XP_010791048.1	20.6	Isocitrate dehydrogenase

Table S9. Proteins less in abundance in *C. rastrospinosus*

Accession Id's	Relative percentage of Total Ion Area	Protein Name
XP_010794573.1	5.7	desmin
XP_010770965.1	2.3	myosin regulatory light chain 2
ACN49202.1	4.7	parvalbumin
XP_010782377.1	11.6	calsequestrin-1
XP_010782071.1	11.1	vitellogenin-2
AAC25100.1	0.4	alpha globin
AAC59671.1	0.4	alpha-1 globin
XP_010794476.1	11.6	betaine--homocysteine S-methyltransferase 1
XP_010771942.1	2.5	vitellogenin-1
XP_010771614.1	15.8	tropomyosin alpha-1 chain isoform X3
XP_010794954.1	9.5	calcium/calmodulin-dependent protein kinase type II subunit beta
XP_010768524.1	14.0	calmodulin
AAC60358.1	3.7	fatty acid binding protein H8-isoform
AAC60372.1	0.0	beta-globin
AAD56588.1	0.0	beta globin
XP_010765488.1	7.8	malate dehydrogenase
XP_010764414.1	1.9	troponin C
XP_010787278.1	12.1	angiotensinogen
XP_010788340.1	13.0	hemopexin
XP_010793059.1	5.7	fibrinogen gamma chain
XP_010778322.1	0.3	haemoglobin subunit zeta
XP_010778161.1	2.0	transmembrane protease serine 2
XP_010771900.1	9.9	fibrinogen beta chain
XP_010785851.1	12.0	protein disulphide-isomerase
XP_010776993.1	5.0	NADP-dependent malic enzyme
CAL92189.1	7.6	transferrin
NP_001290225.1	7.6	serotransferrin precursor
XP_010773398.1	19.8	Calreticulin
XP_010791001.1	26.6	Myosin heavy chain fast skeletal 13
XP_010787188.1	21.1	Ryanodine receptor 1
XP_010790363.1	21.1	Titin-like partial
CAL92189.1	22.7	Transferrin

The differentially expressed proteins in WMM in *C. rastrospinosus* are seen in **supplementary Tables 9 & 10** the more abundant proteins (relative ion area >25) (A) and decreased abundance of proteins (relative ion area <25) (B). Functional enrichment of the proteins showed, more abundant proteins belonging to SRP-dependent co-translational, glycolytic process, and complex V and protein transport. The less abundant protein functional enrichment analysis showed proteins involved in regulation of NADP metabolic process, malate metabolic process, muscle filament sliding, transmembrane transport and isocitrate cellular homeostasis.

Table S10. Proteins more in abundance in *C. rastrospinosus*

Accession Id's	Relative percentage of Total Ion Area	Protein Name
XP_010785279.1	33.2	myosin heavy chain fast skeletal muscle
XP_010776201.1	27.8	titin
AAO24738.1	64.0	creatine kinase muscle isoform 1
XP_010766208.1	56.0	pyruvate kinase PKM
XP_010781933.1	61.4	apolipoprotein B-100
XP_010779868.1	42.2	ATP synthase subunit alpha
XP_010794434.1	42.2	ATP-dependent 6-phosphofructokinase
XP_010785886.1	26.1	nucleoside diphosphate kinase B
XP_010769976.1	44.6	myosin-binding protein H
XP_010764264.1	35.1	elongation factor 2
XP_010774339.1	54.6	ADP/ATP translocase 1
XP_010788727.1	51.1	heat shock protein HSP 90-alpha 1
XP_010780749.1	46.6	malate dehydrogenase
XP_010774927.1	45.5	histone H2AX
XP_010791048.1	35.7	isocitrate dehydrogenase [NADP]
XP_010783416.1	34.3	ryanodine receptor 3
XP_010774282.1	73.6	immunoglobulin and fibronectin type III domain-containing protein 1 isoform X4
XP_010766317.1	34.5	malate dehydrogenase
XP_010782516.1	58.4	voltage-dependent anion-selective channel protein 3
XP_010788362.1	36.4	annexin A1
XP_010789292.1	42.1	eukaryotic initiation factor 4A-I
XP_010793340.1	49.8	obg ATPase 1
XP_010792965.1	33.6	40S ribosomal protein S4
XP_010766151.1	45.2	elongation factor 1-gamma
XP_010773292.1	43.9	dihydrolipoyllysine-residue acetyltransferase component of pyruvate dehydrogenase complex
XP_010771231.1	46.8	T-complex protein 1 subunit alpha
XP_010768032.1	37.1	40S ribosomal protein SA isoform X2
XP_010768026.1	37.1	40S ribosomal protein SA isoform X1
XP_010773064.1	47.8	phosphate carrier protein
XP_010783756.1	39.0	40S ribosomal protein S2
XP_010788327.1	67.6	nebulin
XP_010776310.1	43.7	60S ribosomal protein L9
XP_010794136.1	32.7	ATP synthase subunit g
XP_010793781.1	41.9	phosphoglycerate kinase
XP_010790499.1	32.7	60S ribosomal protein L35
XP_010782543.1	36.3	60S ribosomal protein L6
XP_010765728.1	46.2	ATP synthase subunit beta
XP_010779184.1	48.7	elongation factor 1-alpha
XP_010767902.1	59.4	hyaluronan and proteoglycan link protein 1
XP_010778067.1	49.6	ATP synthase subunit gamma isoform X1
XP_010778068.1	49.6	ATP synthase subunit gamma isoform X2
XP_010789836.1	39.4	60S ribosomal protein L13
XP_010778226.1	49.4	succinyl-CoA ligase [ADP/GDP-forming] subunit alpha

XP_010771562.1	32.6	glycogen debranching enzyme
XP_010786327.1	44.2	ATP synthase F(0) complex subunit B1 mitochondrial
XP_010787537.1	33.0	40S ribosomal protein S8
XP_010784438.1	33.7	ADP/ATP translocase 3
XP_010772744.1	60.2	calcium-binding mitochondrial carrier protein Aralar1
XP_010795693.1	51.1	60S ribosomal protein L4
XP_010777506.1	45.8	NADH-ubiquinone oxidoreductase 75 kDa subunit
XP_010772746.1	82.8	plasminogen activator inhibitor 1 RNA-binding protein isoform X2
XP_010768357.1	58.1	calcium-binding mitochondrial carrier protein Aralar1
XP_010782556.1	33.6	fructose-bisphosphate aldolase A
XP_010795485.1	46.0	fragile X mental retardation syndrome-related protein 1 isoform X3
XP_010772138.1	45.9	ATP synthase subunit O
XP_010784946.1	41.6	T-complex protein 1 subunit zeta
XP_010777740.1	39.1	inter-alpha-trypsin inhibitor heavy chain H3
QBF53709.1	85.1	hemopexin
XP_010766540.1	31.7	glyceraldehyde-3-phosphate dehydrogenase
XP_010769990.1	86.2	heat shock 70 kDa protein 6
XP_010783785.1	27	40S ribosomal protein S12
XP_010765856.1	24	60S ribosomal protein L30
XP_010779104.1	28.5	60S ribosomal protein L12 isoform X2
XP_010790870.1	22.7	Reticulon
XP_010793467.1	22.8	AMP deaminase 1 isoform X1 & X2 [Notothenia coriiceps]
XP_010794693.1	29.1	40S ribosomal protein S13 [Notothenia coriiceps]
XP_010783741.1	25.5	40S ribosomal protein
YP_004581500.1	25.9	Cytochrome c oxidase subunit II
XP_010776714.1	28.4	40S ribosomal protein S25
XP_010779161.1	29.1	60S ribosomal protein L11
XP_010791484.1	29.7	60S ribosomal protein L10a
XP_010786813.1	29.3	40S ribosomal protein S9
XP_010781656.1	28.1	Fructose-1 6-bisphosphatase isozyme 2-like

Table S11. Proteins more in abundance in *C. gunnari*

Accession Id's	Relative percentage of Total Ion Area	Protein Name
XP_010770965.1	Myosin regulatory light chain 2 skeletal muscle isoform-like	94.4
XP_010791910.1	Myozenin-1 isoform X1 & X2	78.9
XP_010788098.1	Heat shock protein beta-1	74.6
XP_010790499.1	60S ribosomal protein L35	56
XP_010773777.1	40S ribosomal protein S16 isoform X1	52.8
XP_010770361.1	60S ribosomal protein L7	52.3
XP_010770797.1	Fragile X mental retardation syndrome-related protein 2	51.6
XP_010783785.1	40S ribosomal protein S12	50.8
XP_010765856.1	60S ribosomal protein L30	50.3
XP_010779104.1	60S ribosomal protein L12 isoform X2	49.8
XP_010790870.1	Reticulon	49.4
XP_010785200.1	Palladin-like	49.2
XP_010776310.1	60S ribosomal protein L9	48.7
XP_010793467.1	AMP deaminase 1 isoform X1 & X2 [Notothenia coriiceps]	48.4
XP_010784438.1	ADP/ATP translocase 3	47.8
XP_010794693.1	40S ribosomal protein S13 [Notothenia coriiceps]	47.3
XP_010787537.1	40S ribosomal protein S8-like partial	46
XP_010791686.1	Alpha-actinin-2	46
XP_010768845.1	Ryanodine receptor 3-like	32.9
XP_010791686.1	Alpha-actinin-2	46
XP_010774286.1	Kelch-like protein 41b	45.7
XP_010782543.1	60S ribosomal protein L6	45.4
XP_010783741.1	40S ribosomal protein S5	45.3
YP_004581500.1	Cytochrome c oxidase subunit II	44.7
XP_010776714.1	40S ribosomal protein S25	44.2
XP_010779161.1	60S ribosomal protein L11	43.7
XP_010767141.1	Voltage-dependent anion-selective channel protein 2	43.5
XP_010764830.1	40S ribosomal protein S26	32.7
XP_010792965.1	40S ribosomal protein S4	43
XP_010783756.1	40S ribosomal protein S2	42.8
XP_010791484.1	60S ribosomal protein L10a	40.4
XP_010786813.1	40S ribosomal protein S9	40.3
XP_010782695.1	ATP-dependent 6-phosphofructokinase muscle type-like	39.9
XP_010777740.1	Inter-alpha-trypsin inhibitor heavy chain H3-like	39.1
XP_010794136.1	ATP synthase subunit g	37
XP_010787927.1	Vinculin	37
XP_010789836.1	60S ribosomal protein L13	36.5
XP_010780163.1	Guanine nucleotide-binding protein subunit beta-2-like 1	36.2
XP_010781656.1	Fructose-1 6-bisphosphatase isozyme 2-like	36.1
XP_010768032.1	40S ribosomal protein SA isoform X2	35.3

XP_010778226.1	Succinyl-CoA ligase	35
XP_010766070.1	Tubulin alpha chain-like isoform X1 & X2	34.9
XP_010791811.1	60S ribosomal protein L23a	34.8
XP_010777506.1	NADH-ubiquinone oxidoreductase	34.7
XP_010765339.1	Alpha-enolase-like	34.5
XP_010791048.1	Isocitrate dehydrogenase	32.6
XP_010777584.1	26S proteasome non-ATPase regulatory subunit 12	30.6
XP_010786327.1	ATP synthase F(0) complex subunit B1	30.2
XP_010790691.1	Peptidyl-prolyl cis-trans isomerase-like	29.5
XP_010780749.1	Malate dehydrogenase	29.4
XP_010772138.1	ATP synthase subunit O	28.6
XP_010785896.1	Glycine-rich RNA-binding protein GRP2A-like	28.4
XP_010776087.1	NAD(P) transhydrogenase	28.3
XP_010788355.1	Glycogen phosphorylase muscle form-like	26.9
XP_010769500.1	Ubiquitin carboxyl-terminal hydrolase 5 isoform X1	26.7
XP_010784571.1	Cytochrome b-c1 complex subunit 2	25
XP_010778067.1	ATP synthase subunit gamma	24.7
XP_010765728.1	ATP synthase subunit beta	23.8
XP_010783619.1	Proteasome subunit alpha type-7-like	23.6
XP_010766216.1	Pyruvate kinase PKM	22.7
XP_010774042.1	FK506-binding protein 1	28.1
XP_010774927.1	histone H2AX	45.5
XP_010794764.1	fibronectin	34.9

The differentially expressed proteins in WMM in *C. gunnari* are seen in **supplementary Tables 11 & 12**. The more abundant proteins (relative ion area >25) (A) and decreased abundance of proteins (relative ion area <25) (B). The increased proteins PPI) had 46 protein nodes with 242 number of edges and a PPI enrichment p-value < 1.0e-16. Functional enrichment of the proteins showed, more abundant proteins belonging Sr-dependent co-translational, TCA, muscle filament, translation initiation, and protein transport.

Table S12. Proteins less in abundance in *C. gunnari*

Accession Id's	Relative percentage of Total Ion Area	Protein Name
XP_010766216.1	22.7	pyruvate kinase PKM
XP_010779868.1	9.8	ATP synthase subunit alpha
XP_010785886.1	13.3	nucleoside diphosphate kinase B
XP_010788727.1	16.1	heat shock protein HSP 90-alpha 1
XP_010764982.1	6.2	myosin-binding protein H
XP_010770129.1	0.5	parvalbumin beta
XP_010774927.1	13.4	histone H2AX
XP_010782377.1	3.9	calsequestrin-1
XP_010782071.1	8.0	vitellogenin-2
XP_010792180.1	9.4	apolipoprotein A-I
XP_010772488.1	14.8	creatine kinase S-type
XP_010769328.1	11.8	phosphoglycerate mutase 2

XP_010786426.1	12.3	sarcalumenin isoform X2
XP_010783416.1	10.6	ryanodine receptor 3
AAC25100.1	3.4	alpha globin
AAC59671.1	3.4	alpha-1 globin
XP_010788362.1	8.1	annexin A1
XP_010771942.1	1.2	vitellogenin-1
AAL99930.1	0.2	IgM heavy chain secretory form
CAL92187.1	2.8	transferrin
XP_010767902.1	4.9	hyaluronan and proteoglycan link protein 1
AAC60372.1	3.4	beta-globin
XP_010765741.1	16.9	heat shock 70 kDa protein
XP_010783999.1	12.0	peroxiredoxin-5
XP_010778322.1	2.6	haemoglobin subunit zeta
XP_010777584.1	11.8	26S proteasome non-ATPase regulatory subunit 12
XP_010792310.1	4.8	actin cytoplasmic 3
XP_010768524.1	18.9	Calmodulin
XP_010773841.1	19.5	40S ribosomal protein S3a
XP_010790854.1	18.1	Obscurin isoform X2

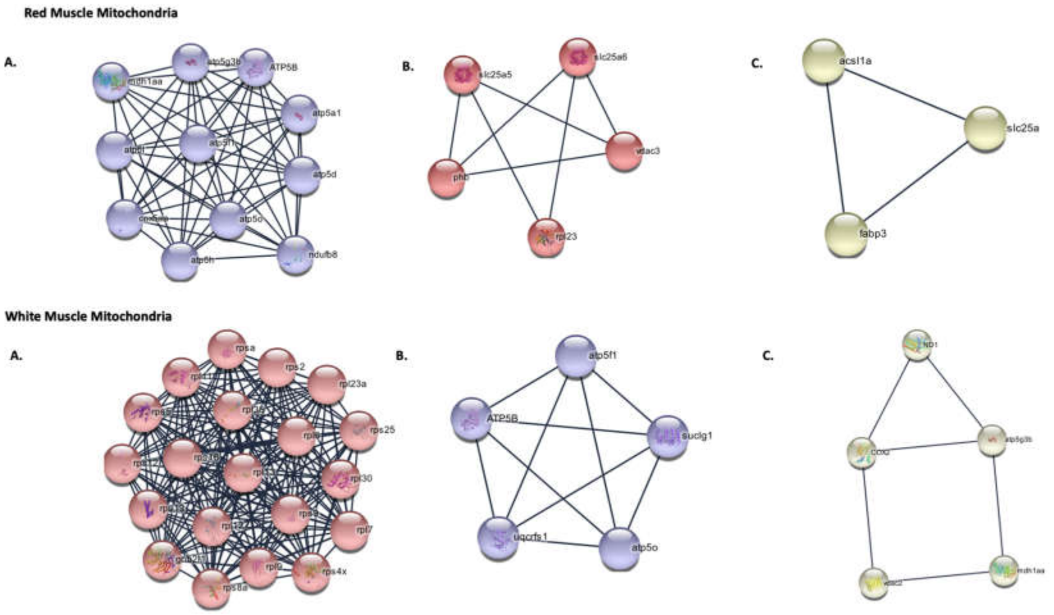

Supplementary Figure S1. Top modules obtained from the PPI network using Cytoscape's plugin MCODE for Red muscle tissue network (C) and white muscle tissue network (D) for upregulated proteins in the icefish in comparison to red-blooded species. There are common modules seen in both RMM and WMM for complex V subunits.

A. Red Muscle Mitochondria (Increase in abundance in icefish)

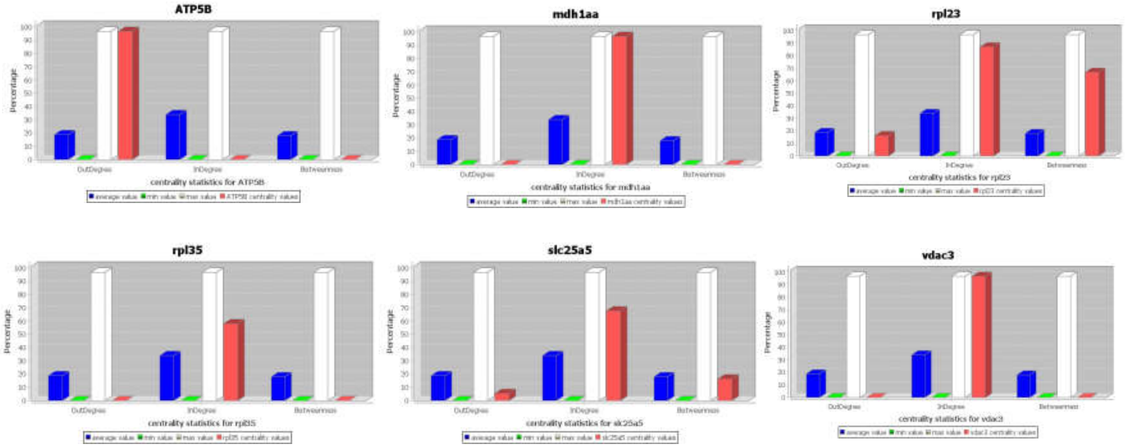

Supplementary Figure S2. Highly connected protein nodes for RMM PPI using CentiScaPe, Cytoscape plugin. ATP5B had the highest percentage of OutDegree whereas MDH1AA has the highest percentage of indegree with VDAC3, RPL23 and SLC25A5 following the trend.

B. White Muscle Mitochondria (Increase in abundance in icefish)

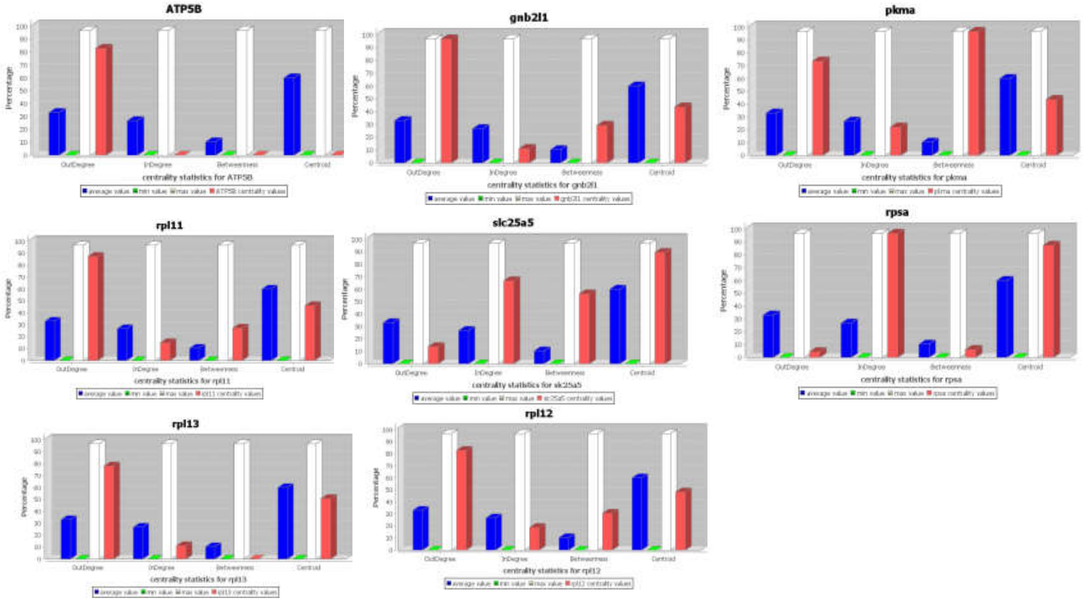

Supplementary Figure S3. Highly connected protein nodes for WMM PPI. Protein GNB2L1 followed by ATP5B (also seen in RMM), RP11 and PKMA have the highest percentage of outdegree. Proteins RPSA, followed by ribosomal proteins RP13 and RP12 and SLC25A5 have the highest percentage of inDegree.

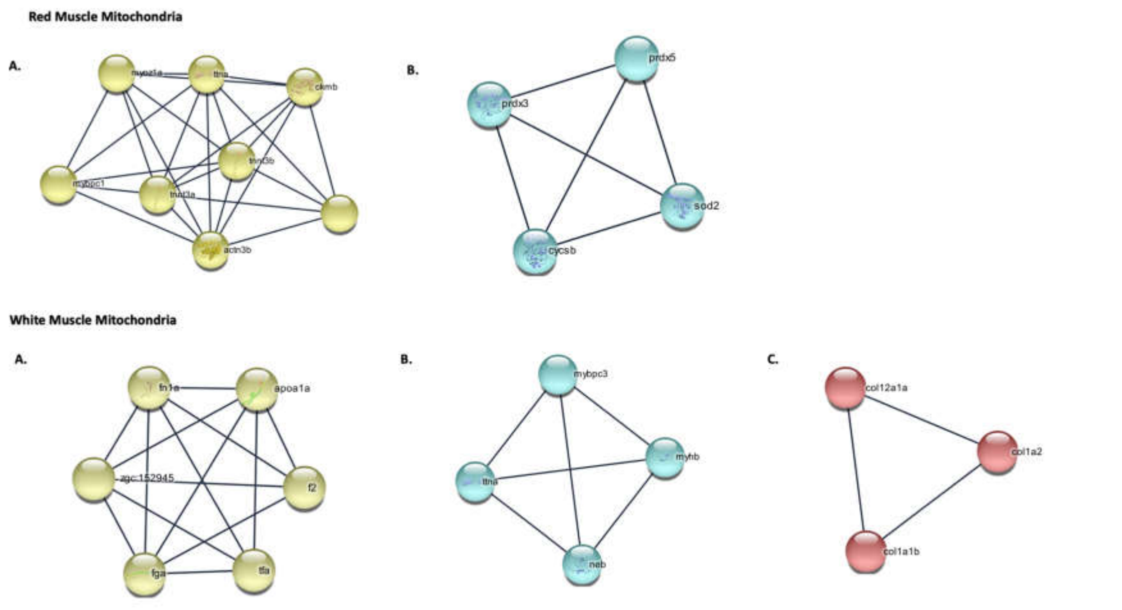

Supplementary Figure S4. Top modules obtained from the PPI network using Cytoscape's plugin MCODE for Red muscle tissue network (C) and white muscle tissue network (D) for upregulated proteins in the icefish in comparison to red-blooded species. Genes with the highest degree of connectivity obtained from the PPI network with Cytoscape.

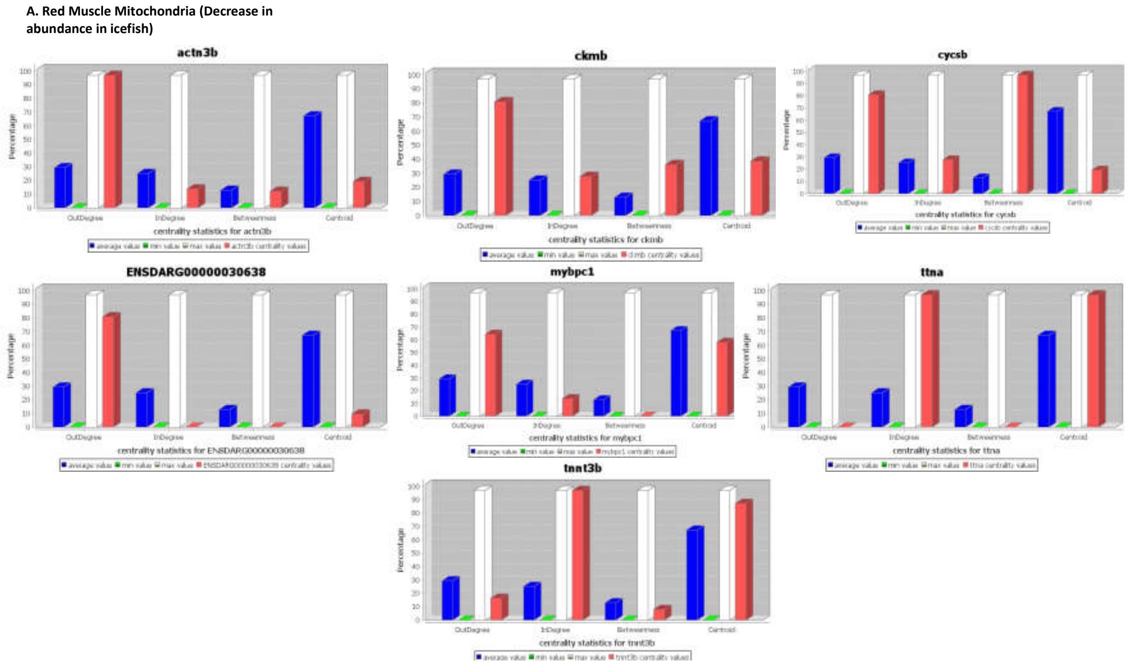

Supplementary Figure S5. Highly connected protein nodes for RMM PPI. Proteins ACTN3B, followed by CKMB, CYSB and MYBPC1 have high percentage of OutDegree. Proteins TTNA and TNNT3B have the highest percentage of InDegree.

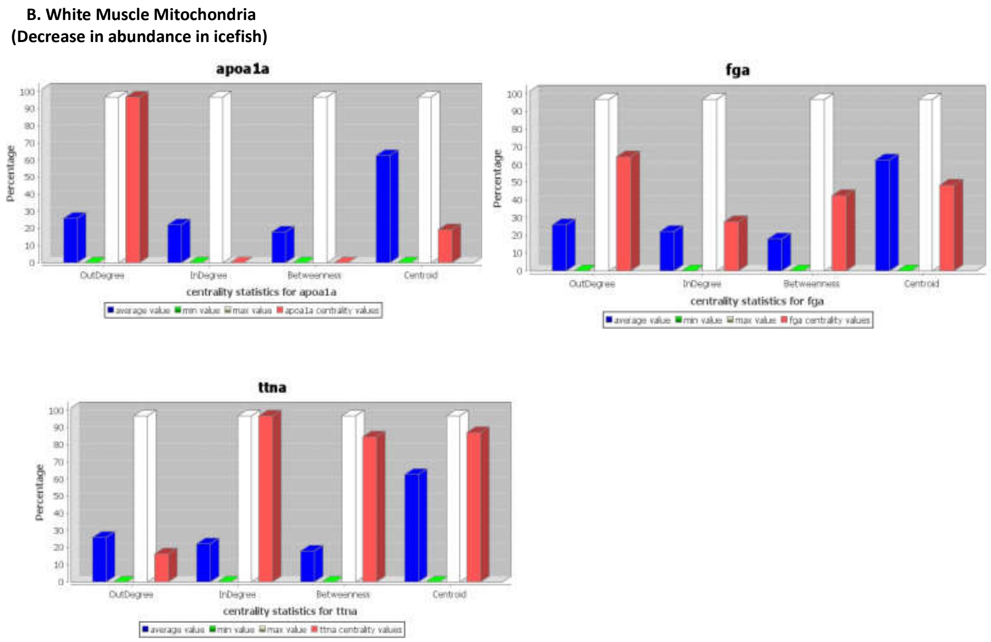

Supplementary Figure S6. Highly connected protein nodes for WMM PPI. Protein APOA1A followed by FGA have the highest percentage of outdegree. Protein TTNA has the highest percentage of inDegree.
